# The impact of poor air quality on hospital attendance of multimorbid patients

**DOI:** 10.3389/fmed.2025.1704117

**Published:** 2026-01-07

**Authors:** Nehal Hassan, Lawin Mohamed, Sarah Wilson, Clare Tolley, Arisha Ahmed, Cait Baxter, Robert Slight, Anil Namdeo, Sarah P. Slight

**Affiliations:** 1 School of Pharmacy, Newcastle University, Newcastle upon Tyne, United Kingdom; 2 Arzheen Pharmacy, Erbil, Iraq; 3 Nottingham University Hospitals NHS Trust, Nottingham, United Kingdom; 4 Northumbria Healthcare NHS Foundation Trust, Newcastle upon Tyne, United Kingdom; 5 Newcastle Upon Tyne Hospitals NHS Foundation Trust Freeman Hospital, High Heaton, Newcastle upon Tyne, United Kingdom; 6 Geography and Environmental Sciences, Northumbria University, Newcastle upon Tyne, United Kingdom

**Keywords:** air pollution, multimorbidity, hospital attendance, healthcare utilisation, health deterioration

## Abstract

**Background:**

Air pollution can severely affect human health. It can contribute to the deterioration of different clinical conditions, leading to increased healthcare utilisation and death. Despite the breadth of evidence on the negative impacts of air pollution on individual long-term conditions, it is currently unclear how air pollution exposure can affect individuals that have different combinations of long-term conditions, and whether it can contribute to hospital attendance. We conducted a systematic review of the literature to understand the impact of air pollution exposure on the hospital attendance of multimorbid patients.

**Methods:**

We searched six major databases (Medline via Ovid, Embase via Ovid, Web of Science, CINAHL, Global Health, and Scopus) using grouped MeSH terms, including “air pollution”, “multimorbidity”, “association” and “hospitalisation” with no time restrictions. Articles published in English that evaluated the impact of various air pollutants (PM_2.5_, PM_10_, SO_2_, NO_2_, O_3_, and CO) on hospital attendance were included. The review is registered with The International Prospective Register of Systematic Reviews (CRD42022369757) and followed the Preferred Reporting Items for Systematic Reviews and Meta-Analyses (PRISMA) guidelines.

**Results:**

Nineteen studies met the inclusion criteria. We categorised them into four clinical groups (“cardiovascular,” “respiratory,” “neurodegenerative and mental health,” and “dependence and hepatic diseases”) based on the primary diagnosed condition of patients in these studies. Although PM_2.5_ was the most studied air pollutant in relation to hospital attendance, NO_2_ lead to an increase in hospital attendance of multi-morbid patients after a relatively short exposure period (2 days), when compared to PM_2.5_ (5 days). We found that factors relating to Climatic conditions (e.g., temperature), Air Pollutants, Demographic factors (e.g., age, biological sex) and Chronic long-term conditions (the “CADC” effect) can influence the likelihood of hospital attendance amongst multi-morbid individuals in the same group.

**Conclusion:**

Exposure to air pollutants increased the likelihood of hospital attendance among multimorbid patients. Future research is required to understand the CADC effect particularly among those from lower socio-economic backgrounds.

**Systematic review registration:**

International prospective register of systematic reviews (CRD42022369757).

## Introduction

Air pollution is defined as environmental contamination by a physical, chemical or biological substance modifying the natural structure of the atmosphere (1). Air pollution is ranked the fourth global health risk to human health and mortality worldwide (1). According to the World Health Organisation (WHO), 99% of the world population breathes air that exceeds the recommended pollution levels, contributing to 7 million deaths annually (1).

There are five main air pollutants that can have an impact on human health: particulate matter (PM), carbon monoxide (CO), sulphur dioxide (SO_2_), nitrogen dioxide (NO_2_), and ozone (O_3_) (2, 3). The size of PM is important, as fine particles (0.1 to 2.5 micrometers) can penetrate the lung and distribute through the blood stream, reaching different body organs (4). Short-term exposure to low levels of PM (defined as 3–5 days) can exacerbate chronic conditions such as Chronic Obstructive Pulmonary Disease (COPD) and asthma, leading to an increase in emergency visits and hospitalisation (5). Short-term exposure to high levels of NO_2_ has also been associated with an increased likelihood of mental health conditions such as depression (6). Long-term exposure (defined as >1 year) to high levels of NO_2_ has been associated with high mortality risk in COPD patients (7). Long-term exposure to high levels of a combination of both PM and NO_2_ have been associated with increased odds of developing chronic diseases, such as diabetes mellitus (8). By 2035, disease cases associated with exposure to NO_2_ alone will have cost the NHS an estimated £9.1 billion (9); consequently, there is a push to lower PM_2.5_ and NO_2_ levels by a minimum of 1 μg/m^3^ by the year 2035 and save NHS resources (9).

Almost 15% of the population in England have two or more chronic conditions (i.e., multi-morbid), Valabhji et al. (10) which is associated with increased healthcare utilisation and costs (11). Despite the breadth of evidence on the negative impacts of air pollution on individual long-term conditions, it is current unclear how air pollution exposure can affect multi-morbid individuals with different combinations of long-term conditions and whether it can contribute to hospital attendance. We conducted a systematic review to understand the impact of short- and long- term exposure to common air pollutants on the hospital attendance of multi-morbid patients.

## Methods

This systematic review followed the Preferred Reporting Items for Systematic Reviews and Meta-Analyses (PRISMA) guidelines, Page et al. (12) and was registered with the International Prospective Register of Systematic Reviews (PROSPERO) (CRD42022369757). The PRISMA checklist is available in Appendix 1 in supplementary material.

### Eligibility criteria

We adopted Population, Intervention, Comparison, Outcome and study design (PICOS) framework to set the inclusion and exclusion criteria for this review. Table 1 demonstrates our PICOS criteria. We included studies that investigated the impact of short- and long-term exposure to air pollutants on the hospital attendance of adult multimorbid patients. At least one of the following air pollutants: PM_2.5,_ PM_10_, SO_2_, NO_2_, O_3_, or CO had to be considered in included articles (2). We defined “hospital attendance” as any unplanned admission or emergency department visit to a secondary or tertiary care centre. Multimorbidity was defined as having two or more chronic conditions at the time of attendance. Studies were also eligible for inclusion if authors specified that patients had both primary and secondary diagnoses. Peer-reviewed articles published in English or with an English language version were eligible for inclusion. Any articles that focused on the impact of poor air quality exposure on children or non-multimorbid patients, and/or articles that were not peer-reviewed studies (i.e., conferences proceedings, editorials, commentaries) were excluded.

**Table 1:** Inclusion and exclusion criteria according to PICOS framework.

PICOS criteria	Inclusion	Exclusion
Population	Adults with two or more long term conditions	Healthy adults, or those with one long-term condition and children.
Intervention	Exposure to one or more of six air pollutants (PM2.5, PM10, SO2, NO2, O3, or CO)	Exposure to any other different air pollutant or any other environmental hazard.
Comparator	Not applicable	
Outcome	Hospital attendance (any unplanned admission or emergency department visit to a secondary or tertiary care centre)	Planned hospital admission (i.e., elective admission) or no hospitalisation.
Study design	Peer-reviewed, quantitative studies (i.e., randomised controlled trials, retrospective research, cohort studies.	Non-Peer-reviewed articles, case series or case studies.

### Information sources and search strategy

Six large databases, including Medline (via Ovid), Embase (via Ovid), Web of Science, Cumulative Index to Nursing and Allied Health literature (CINAHL), Global Health, and Scopus were searched on 14th June 2023 from the date of commencement of the databases. Keywords were grouped into four sets guided by the review question and inclusion criteria: air pollution, association, hospital attendance, and multimorbidity. The four sets were combined using Boolean operators (i.e., “AND,” “OR”). A full list of keywords can be found in Appendix 2 in supplementary material. All the retrieved titles were exported to EndNote (version 20, Clarivate, Jersey, United States), a reference management tool, and duplicates identified and removed (13, 14).

### Study selection

**Figure 1:**
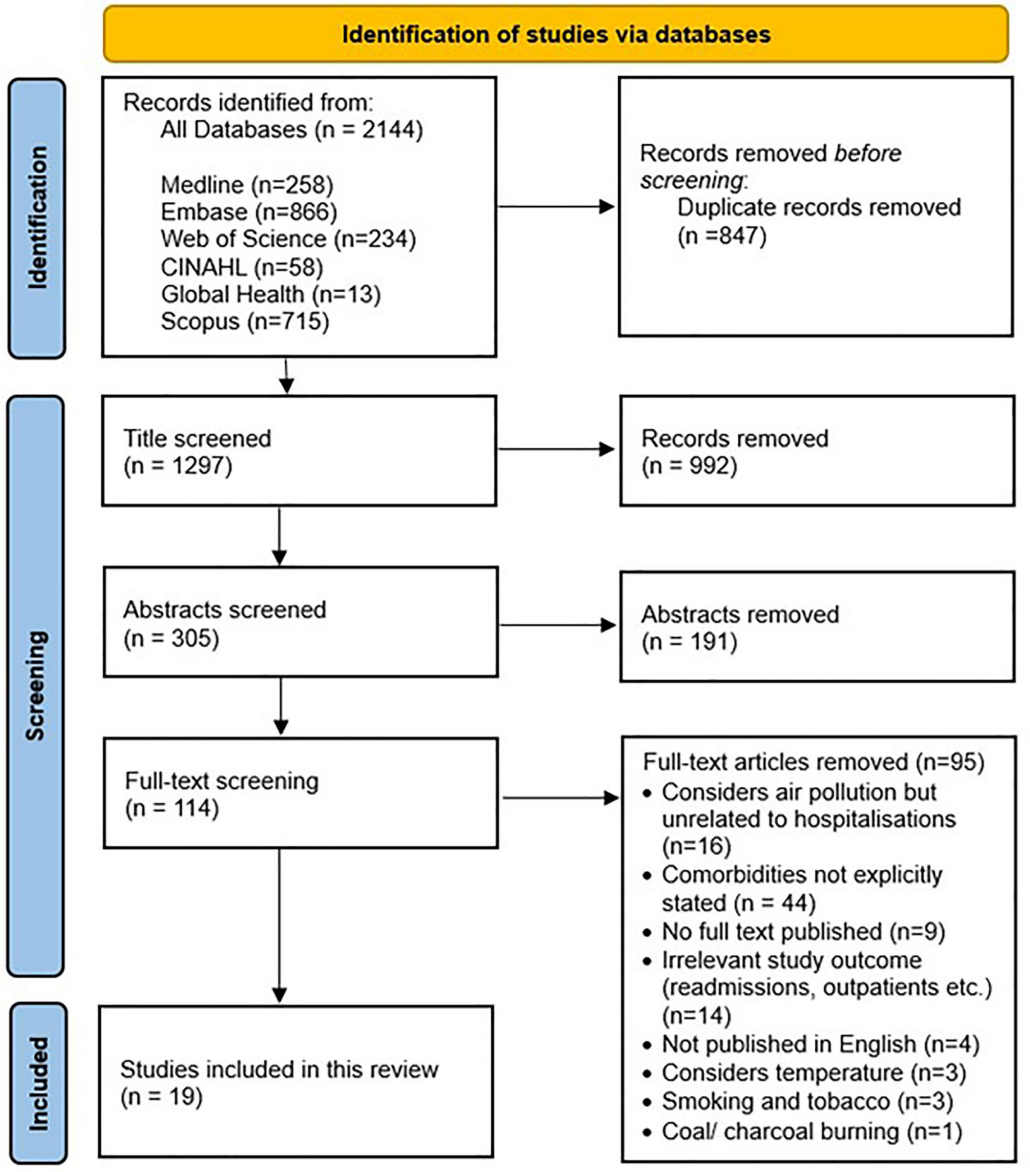
PRISMA flowchart.

Titles, abstracts and full-texts were screened by three independent reviewers (LM, AA, CB). Any disagreements were highlighted and resolved by discussion. Inter-rater reliability among the independent reviewers was 100%. References of the included full-text studies were also screened to identify any additional articles. Figure 1 shows the search strategy through PRISMA flow chart.

### Data extraction

A bespoke data extraction form was developed and used to extract key information from the included studies, including a description of the study (i.e., authors, country, citation, year of publication), study design (i.e., prospective, cohort, retrospective), age of participants, information about multimorbidity (type of multimorbidity, number of chronic conditions), information about the air pollutant (i.e., type, duration of exposure), hospital attendance (type of attendance, length of stay). Data extraction form can be found in Appendix 3 in supplementary material.

### Assessment of the risk of bias and evidence appraisal

The Critical Appraisal Skills Programme (CASP) checklist tool for cohort studies was used to critically appraise the quality of the included studies (15, 16). The CASP checklist consisted of 12 questions divided on three sections; these sections evaluated the validity of the study results, assessment of the results precision, and if the results could be applied to the local population. Detailed responses to the CASP checklist are in Appendix 4 in supplementary material. The risk of bias assessment has also been assessed using the Newcastle Ottawa Scale (NOS). The NOS assess the quality of nonrandomised studies with its design, content and ease of use. It uses a “star system” in which a study is judged on three aspects: the selection of the study groups; the comparability of the groups; and the ascertainment of either the exposure or outcome of interest for case–control or cohort studies, respectively (17). Appendix 5 in supplementary material shows detailed NOS scoring and interpretation of the included articles.

## Results

### Studies description and characteristics

A total of 2,144 articles were extracted from six large databases: Medline (*n* = 258), Embase (*n* = 866), Web of Science (*n* = 234), CINAHL (*n* = 58), Global Health (*n* = 13) and Scopus (*n* = 715). After duplicates (*n* = 847) were removed, articles were screened at the title (*n* = 1,297), abstract (*n* = 305) and full-text stages (*n* = 114); 19 articles met our inclusion criteria (18–36). These studies were conducted in China (*n* = 7) (19, 21–23, 25, 26, 29), United States (*n* = 3) (18, 34, 36), Taiwan (*n* = 3) (20, 28, 33), Denmark (*n* = 1) (34), Iceland (*n* = 1) (31), Italy (*n* = 1) (35), Japan (*n* = 1) (24), South Korea (*n* = 1) (27), and Spain (*n* = 1) (30). All included studies had a retrospective study design, with eight case-cross studies (18, 20, 24, 26, 30, 33, 34, 35), and 11 cohort studies (19, 21–23, 25, 27–29, 31, 32, 36). Table 2 describes the source of included studies.

**Table 2:** Description for the sources of the included studies.

Included study authors	Journal	Indexing databases	Year of publication	Country
Chen et al. (18)	Plos ONE	Medline, Scopus, Web of Science, EMBASE	2022	United states
Li et al. (19)	Environment International	Medline, Scopus, Web of Science, EMBASE	2022	China
Lin et al. (20)	BMJ Open	Medline, Scopus, Web of Science, EMBASE, CINAHL	2022	Taiwan
Liu et al. (21)	Ecotoxicology and Environmental Safety	Medline, Scopus, Web of Science, EMBASE	2021	China
Liu et al. (22)	Ecotoxicology and Environmental Safety	Medline, Scopus, Web of Science, EMBASE	2021	China
Chen et al. (23)	Environmental Science and Pollution Research	Scopus, Web of Science	2020	China
Seposo et al. (24)	Science of The Total Environment	Scopus, Web of Science, EMBASE	2020	Japan
Liu et al. (25)	Atmospheric Environment	Scopus, Web of Science, EMBASE	2020	China
Wang et al. (26)	Environment international	Medline, Scopus, Web of Science, EMBASE	2018	China
Lee et al. (27)	Scientific reports	Medline, Scopus, Web of Science, EMBASE	2017	South Korea
Cheng et al. (28)	Ophthalmology	Medline, Scopus, Web of Science, EMBASE	2016	Taiwan
Zhang et al. (29)	Journal of Epidemiology	Medline, Scopus, Web of Science	2016	China
Alvaro-Meca et al. (30)	Journal of the International AIDS Society	Medline, Scopus, Web of Science, EMBASE, Global Health	2015	Spain
Carlsen et al. (31)	Environmental health	Medline, Scopus, Web of Science, EMBASE, Global Health	2013	Iceland
Anderson et al. (32)	American journal of respiratory and critical care medicine	Medline, Scopus, Web of Science, EMBASE, CINAHL	2011	Denmark
Cheng et al. (33)	Inhalation toxicology	Medline, Scopus, Web of Science, EMBASE	2009	Taiwan
Peel et al. (34)	American Journal of Epidemiology	Medline, Scopus, Web of Science, EMBASE, Global Health	2007	United states
D’Ippoliti et al. (35)	Epidemiology	Medline, Scopus, Web of Science, EMBASE	2003	Italy
Zanobetti et al. (36)	American journal of respiratory and critical care medicine	Medline, Scopus, Web of Science, EMBASE, CINAHL	2001	United States

Multimorbid patients included in these studies had a range of different long-term conditions, including asthma (20, 21, 33, 34), Chronic Obstructive Pulmonary Disease (COPD) (20, 24–26, 31–34), diabetes mellitus (21, 22, 25–29, 31, 33–36), heart failure (18, 24–27, 33, 34), hypertension (23, 25, 28, 29, 33–35), stroke (22, 31, 33), dementia (27), coronary heart disease (28, 33), Human Immune Deficiency virus (HIV) (30), dysrhythmia (28, 33–35), liver disease (30), cancer (30), and depression (26). We grouped the patients into “multimorbidity groups” depending on their primary diagnosis (condition that occurred first in the patient). For example, if the patient had a primary diagnosis of heart failure or hypertension, they were included in the cardiovascular group. Twelve studies were grouped in the cardiovascular group (18, 19, 22–25, 28, 29, 33, 34, 35), four studies in the respiratory group (20, 21, 32, 36), two studies in the neurodegenerative and mental health group (26, 27), and one study in the Dependence and Hepatic disease (30). Each included study only focused on one specific theme and was grouped accordingly. Table 3 describes the primary condition and co-morbidities in each study.

**Table 3:** Primary condition and co-morbidities in each study.

Study	Group	Primary condition	Co-morbidity
Chen et al. (18)	Cardiovascular	HF	Unspecified
Li et al. (19)	Cardiovascular	CVD	Diabetes
Lin et al. (20)	Respiratory	Asthma	COPD
Liu et al. (21)	Respiratory	Asthma	Diabetes
Liu et al. (22)	Cardiovascular	Stroke	Diabetes
Chen et al. (23)	Cardiovascular	HTN	stroke, COPD or CV
Seposo et al. (24)	Cardiovascular	HF	COPD
Liu et al. (25)	Cardiovascular	HF	HTN, Diabetes, COPD
Wang et al. (26)	Neuro degenerative and Mental Health	depression	HF, Diabetes, COPD
Lee et al. (27)	Neuro degenerative and Mental Health	Parkinson’s	Dementia (12%), Diabetes (12%), Cerebral infarction (9%)
Cheng et al. (28)	Cardiovascular	HTN	Diabetes
Zhang et al. (29)	Cardiovascular	HTN	Diabetes
Alvaro-Meca et al. (30)	Dependence/Hepatic	HIV	liver disease, chronic pulmonary disease, cancer
Carlsen et al. (31)	Cardiovascular	HF	Stroke, COPD, Diabetes
Anderson et al. (32)	Respiratory	COPD	Unspecified
Cheng et al. (33)	Cardiovascular	HTN	Asthma, COPD, Diabetes, HF
Peel et al. (34)	Cardiovascular	CHF	HTN, Diabetes, asthma, dysrhythmia, COPD, athersclerosis
D’Ippoliti et al. (35)	Cardiovascular	Hypertension	Arrhythmia, Diabetes
Zanobetti et al. (36)	Respiratory	COPD	Diabetes

### Hospital attendance amongst patients in the cardiovascular group

Twelve studies investigated the impact of six air pollutants (PM_2.5_, PM_10_, SO_2_, NO_2_, O_3_, and/or CO) on hospital attendance in multimorbid patients whose primary diagnosis was a cardiovascular (CV) condition and who also had a second condition such as diabetes (18, 19, 22–25, 28, 29, 31, 33–35).

#### Short term exposure (between 2 and 14 days)

Two-day exposure to PM_10_, NO_2_, CO, or SO_2_ were linked to higher emergency department (ED) visits among multi-morbid patients with a combination of several diagnosed CV conditions, when compared with those without multimorbidity (34). Multi-morbid patients with a CV condition (e.g., hypertension), diabetes and a third condition of COPD showed higher emergency admissions with rising levels of CO or SO_2_, compared to those without COPD (34).

In another study, Chen et al. (23) patients with primary condition as hypertension and stroke, COPD or CV comorbidities were at higher risk of AMI hospital admissions when exposed to increased levels of PM_2.5_, PM_10,_ SO_2_, NO_2_, and/or O_3_ for 5 days (23). Of these patients, those having stroke as a comorbidity demonstrated higher stroke hospital attendance when exposed to high levels of SO_2_ for 4 days (1.124; 95% CI, 1.015–1.245) and PM_2.5_ for 3 days (1.012; 95% CI, 1.003–1.096), compared to non-hypertensive patients. Also, the risk of AMI hospital attendance was generally higher with the 5-days exposure to PM_2.5_, PM_10_ and/or NO_2_ among those with a coronary artery disease comorbidity versus those who have not (23).

Five-day exposure to PM_2.5_ was specifically associated with an increase in hospital attendance rates of 2.5% (95% CI: 0.7, 4.4%) for multi-morbid patients with a combination of ischemic heart disease (IHD) and congestive heart failure (CHF), and 1.7% (95% CI: 0.6, 2.8%) for those with a combination of IHD and hypertension (22). For the later, this increase was 1.8 times higher for these multi-morbid patients than for non-hypertensive patients (22). Another study showed an increase in ED visits amongst multi-morbid patients who had a combination of heart failure and COPD after 6 days of exposure to high levels of NO_2_ and 14 days of exposure to O_3_ (24).

Seven-day exposure to increased levels of PM_2.5_, PM_10_, O_3_, and/or CO was associated with an increased rate of hospital attendance for multi-morbid patients in this CV group (19). Multi-morbid patients who had both CV disease and diabetes were also found to have stayed longer in hospital (19). There was a slight increase in hospital attendance rates of 0.53% (95% CI: 0.45 to 0.61), 0.98% (95% CI: 0.81 to 1.16) and 0.93% (95% CI: 0.67 to 1.20) with every 10 μg/m^3^ increase in O_3,_ SO_2_, and NO_2_ over the 7days, respectively (19).

#### Long-term exposure (over 1 year)

Long-term exposure to PM_2.5_ (>1 year) in multi-morbid patients who had a primary diagnosed CV condition (e.g., heart failure) and a second diagnosed condition such as diabetes, hypertension or coronary heart disease were moderately associated with increased hospital attendance, with every 1 μg/m^3^ increase in PM_2.5_ (18).

#### Demographic factors (age and biological sex)

Age and sex appeared to be risk factors for increased hospital attendance amongst patients in the CV group with concurrent rises in short-term air pollution exposure (31). Female patients with CV disease and pulmonary multimorbidity had a 7.8% increase in cardiopulmonary hospital attendance, when exposed to an increase of 12.93 μg/m^3^ in NO_2_ levels 2days prior (31). Similarly, older patients (≥70 years) with the same long-term conditions demonstrated a 3.9% increase in cardiopulmonary hospital attendance when exposed to the same short-term increase (31). In this study, females with a primary CV conditions and a second pulmonary condition had a higher stroke hospital attendance rate (7.8%) when compared to males (3.5%), with an overall increase of 3.9% in hospital attendance rate with every IQR increase of 17.21 μg/m^3^ in average O_3_ (31). In another study, males <65 years old with primary condition as hypertension and stroke comorbidity, appeared to be more susceptible than the rest of the population to stroke hospital attendance when exposed to O_3_ at 2 days exposure (1.178, 95%CI, 1.002–1.352) (23).

Multi-morbid patients who had been diagnosed with a primary condition of stroke and who also had a secondary condition of diabetes were more likely to be admitted for ischemic stroke 0.14% (95% CI: 0.06–0.22%) and haemorrhagic stroke 0.72% (95% CI: 0.02–1.42%) when exposed to a 10 μg/m^3^ increase in PM_2.5_ levels for 2 days (22). This association was also influenced by age, but with higher effect estimates reported in younger adults (18–65 years). The authors hypothesised that older participants may pay more attention to preventing environmental hazards from PM pollution. For example, older participants may have more flexibility to stay at home during heavy pollution days (22). Females were found to have a slightly higher increase in haemorrhagic stroke admissions (0.15%) when exposed to high levels of PM_10_, compared to males (0.14%) (22).

In another study, multi-morbid patients who had been diagnosed with hypertension as a primary condition, and one of the following conditions: diabetes, coronary artery disease, hyperlipidaemia, cerebral infarction, arrhythmia, heart failure, carotid artery stenosis, rheumatic heart disease, or glaucoma were associated with an increased risk of central retinal artery occlusion (CRAO) admissions when exposed to PM_2.5_, PM_10,_ SO_2_, NO_2_, or O_3_ for up to 5 days (28). However, this association varied with the type of pollutant, health condition(s) and age. For example, patients (≥65 years) who had a primary diagnosis of hypertension and another condition (diabetes) demonstrated a significant association with CRAO admissions at 4 days of exposure to both NO_2_ and SO_2_ (OR, 1.40; 95% CI, 1.05–1.87). In contrast, the associations did not reach statistical significance with other air pollutants (28). For acute myocardial infarction (AMI) hospital attendance, hypertensive patients with diabetes comorbidity exposed to a 1 day increase of 10 μg/m^3^ in PM_2.5_ had increased rates of ST-elevation myocardial infarction (STEMI) (OR 1.05; 95% CI, 1.00–1.11), particularly among male patients over 65 years old (29). However, this association was not observed with overall AMI (unstable angina and NSTEMI) (29).

#### Climatic conditions (temperature)

Multi-morbid patients who had been diagnosed with a primary condition of hypertension and who also had a second or third health condition of diabetes and hypertension or asthma, demonstrated an increased likelihood of AMI hospital admissions, when exposed to a 10 μg/m^3^ increase in CO or NO_2_ up to 2 days before admission (35). This association appeared to be stronger in warmer weather amongst those who had heart conduction problems (i.e., arrhythmias) and older adults (>74 years) (35).

In another articles, the impact of up to 5 days of exposure to PM_10_, SO_2_ or CO on pneumonia hospital attendance in patients with a primary diagnosed condition of a CV condition such as hypertension, dysrhythmia, cerebrovascular disease, CHF, or IHD and a comorbid pulmonary condition [COPD, asthma or upper respiratory tract infection (URI)] was examined (33). There was difference in hospital attendance based on the temperature and comorbidity (33). During warm weather (a mean daily temperature above 25 °C), there was a 28% increase in pneumonia hospital attendance due to an exacerbation of URI when exposed to an increase of 61.94 ug/m^3^ in PM_10_, compared to a 23% increase in those without URI. Similarly, with an increase in CO levels, there was 31% increase in hospital attendance among those with URI versus 24% in non-URI patients (33). During cool weather (mean daily temperature below 25 °C), these associations appeared to be even higher with a similar IQR increase in PM_10_ and CO resulting in a 70 and 64% increase in admissions amongst those with URI, respectively, and compared to 64 and 55% in non-URI patients, respectively (33).

### Hospital attendance amongst patients in the respiratory group

Four studies investigated the impact of four air pollutants (PM_2.5_, PM_10,_ NO_2_, and/or O_3_) on the hospital attendance of multimorbid patients who had a primary diagnosis of a respiratory condition (Asthma or COPD) (See Table 3) (20, 21, 32, 36).

#### Short-term exposure

Multi-morbid patients who had been diagnosed with a primary condition of COPD, and who also had diabetes or CV comorbidities were more likely to attend hospital with a 10 μg/m^3^ increase in PM_10_ exposure over the course of 1 day (36). These multi-morbid patients showed an increase of 2.01% (95% CI 1.40–2.62%) in CVD admissions, 4.37% (95% CI − 0.76-5.44%) in COPD admissions, and 2.77% (95% CI 1.20–4.37%) in pneumonia hospital attendance, compared to 0.94, 1.5 and 2.2% for CVD, COPD and pneumonia hospital attendance in individuals with COPD alone (36). In another study, multi-morbid patients who had been diagnosed with a primary condition of asthma and had different comorbidities (e.g., CVD, diabetes, COPD, renal disease, liver disease or peptic ulcer or neurological conditions) had an increased hospital attendance after being exposed to increased O_3_ levels over 1 day (20). In this study, patients who had a combination of both asthma and COPD disease, and who were 65 years or above, were most affected (20). Furthermore, patients who had a group of health conditions including respiratory and diabetes disease had a statistically significant increase (2.16%) in respiratory disease hospital attendance after 8 days exposure to increased level of PM_2.5_, compared to a 1.92% increase in respiratory patients who did not have diabetes (*p*-value <0.001) (21).

#### Long-term exposure

Multi-morbid patients who had been diagnosed with COPD as their primary condition and had diabetes or asthma comorbidities were strongly associated with an increase in COPD hospital attendance (OR 1.29 diabetes) or asthma hospital attendance (OR 1.19 asthma), respectively, after being exposed to a 35-year weighed mean NO_2_ level of 18.1 ± 5.6 μg/m^3^ (hazard ratio, 1.08; 95% CI, 1.02–1.14, per IQR 5.8 μg/m^3^) (from traffic emissions) (32).

### Hospital attendance amongst patients in the neurodegenerative and mental health group

Two studies were included in this group (26, 27). One study investigated the impact of PM_2.5_ and PM_10_ on the hospital attendance of multimorbid patients who had depression as their primary diagnosis and another condition such as heart failure, diabetes or COPD (26). A positive correlation was found between five-days exposure to PM_2.5_ and PM_10_ and increasing hospital attendance amongst patients with depression and cardiovascular diseases broadly (26). This study also considered demographic factors (age) and found an increase in hospital attendance amongst older people (>65 years) of 9.23% (IQR: 5.09–13.53) and 6.35% (IQR: 3.31–9.49) with increased levels of PM_2.5_ and PM_10_, respectively; this was compared to a 1.49% (IQR: 0.25–3.27) and 1.32% (IQR: 0.04–2.61) increase in hospital attendance in younger patients (<65 years) with depression as primary diagnosis (24).

Among multi-morbid female patients (65–74 years) with Parkinson’s as a primary condition and a second condition of dementia, diabetes or cerebral infarction, there was a significant association (*p* < 0.05) between hospital attendance due to Parkinson’s aggravation and exposure to increased levels of PM_2.5_, SO_2_, NO_2_, O_3_, or CO for 8 days (27).

### Hospital attendance amongst patients in the dependence and hepatic disease group

We labelled this group based on evidence from existing literature establishing associations between alcohol dependence and drug misuse which are strongly linked to hepatic diseases, particularly with intravenous drug abuse (37–41). Only one study examined the impact of CO, PM_10_, NO_2_ and O_3_ on the hospital attendance of multimorbid patients who had HIV as their primary diagnosis (See Table 3). This study investigated pneumocystis pneumonia (PCP) hospital admissions in patients who were HIV-positive and also had hepatic, chronic pulmonary disease or cancer (30). Exposure to higher levels of PM_10_ and NO_2_ at four different time points (two, four, six and eight weeks) was found to be significantly associated with an increase in PCP hospital attendance (*p* < 0.001). Higher concentrations of O_3_ in particular were significantly associated with PCP admissions at the 1 month time-point (*p* = 0.007), one and half month time-point (*p* < 0.001), and 2 month time-point (*p* = 0.006), while CO was only significantly associated with PCP admissions at the 8 week time-point (*p* < 0.001) (15). This study also considered climatic conditions (temperature) and reported that the highest rate of hospital attendance was in winter, while the lowest rate was recorded during summer months (30).

## Discussion

This is the first systematic review to explore the impact of exposure to six common air pollutants (PM_2.5_, PM_10_, NO_2_, CO, SO_2_, and/or O_3_) on hospital attendance of multimorbid patients. Studies were grouped in four groups (cardiovascular, respiratory, liver and dependence, and mental health) and informed by previous literature (42). Multi-morbid patients who had a CV condition as their primary diagnosis (CV group) and another condition were the most studied group. PM_2.5_ and PM_10,_ NO_2_ and O_3_ had the highest influence on hospital attendance among different groups. The short-term exposure duration ranged from between 2 and 5 days, and the long-term exposure was studied up to 35 years, particularly for COPD admissions. Exposure to different combinations of air pollutants increased the rate of hospital attendance amongst multimorbid patients in the respiratory group. Multi-morbid patients in Cardiovascular and respiratory groups were more likely to have increased hospital attendance with short-term exposure to PM_2.5_ and PM_10_.

This review demonstrated how the likelihood of hospital attendance may vary within multimorbidity groups. People in the CV group were at the highest risk of hospital attendance when exposed to different air pollutants for a duration as short as 2 days (34). Although PM_2.5_ was the most studied air pollutant in relation to hospital attendance (18, 19, 22, 28, 33). NO_2_ lead to an increase in hospital attendance after a relatively short exposure period (2 days) (23, 34, 35), when compared to PM_2.5_ (5 days) (22, 28). NO_2_ is one of the most prevalent air pollutants in deprived communities. In England, NO_2_ level was 6 μg/m^3^ higher in the most deprived areas when compared to levels in the least deprived areas (43). Another study also found NO_2_ levels to be higher in the most deprived areas of England compared to least deprived ones, with this difference far smaller with PM_10_ (44). These figures represent an urgent need to take action to reduce the exposure to NO_2_ levels and subsequent hospital attendance.

**Figure 2:**
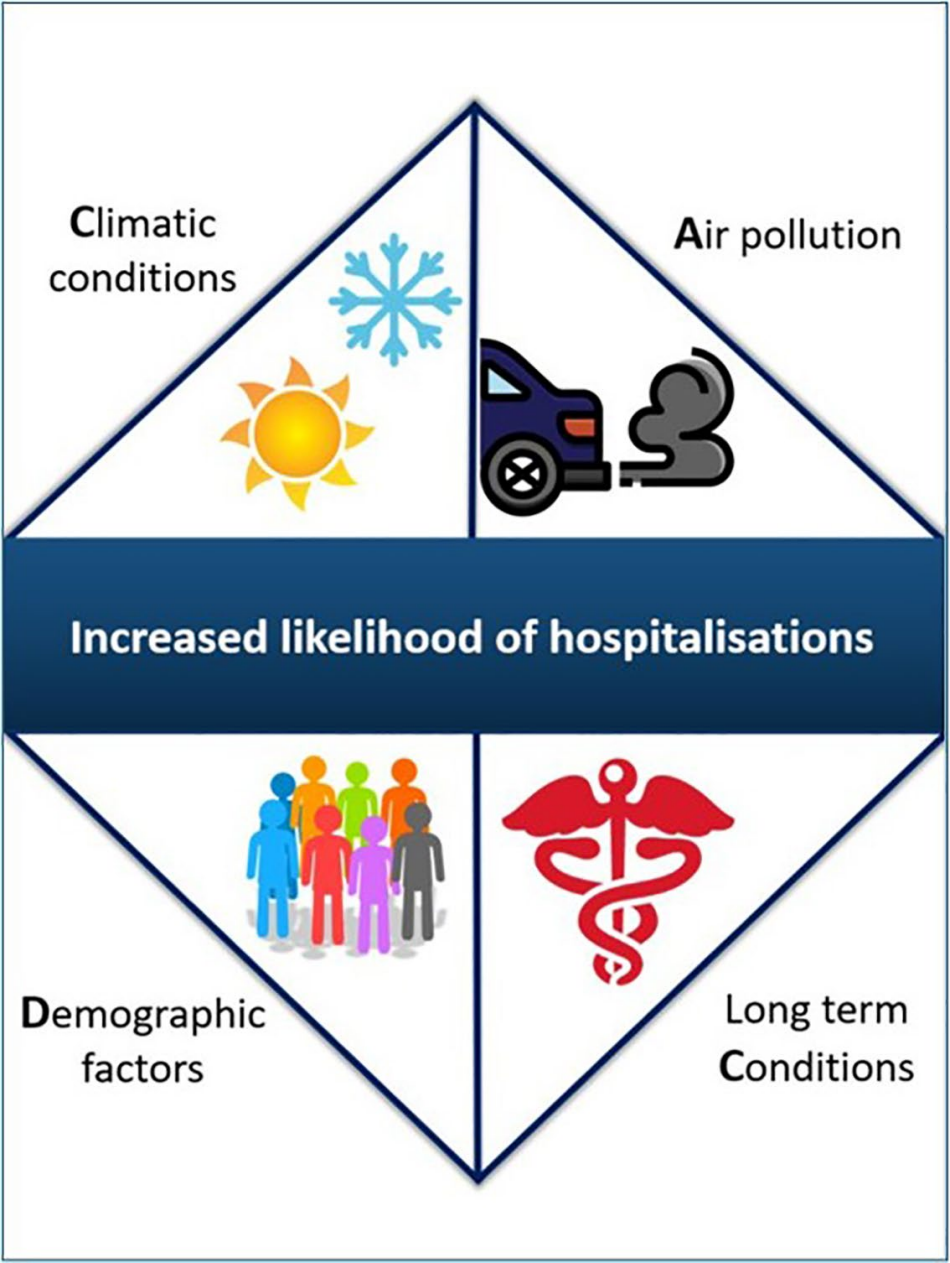
‘CADC’ effect (Climatic, Air pollutants, Demographics, and the type of Chronic conditions).

This systematic review also revealed how some patients were more vulnerable than others to air pollution exposure within multi-morbidity groups, due to other factors such as age, biological sex, and temperature. We named this the “*CADC*” effect which took into account Climatic, Air pollutants, Demographics, and the type of Chronic conditions (see Figure 2).

The most prominent *demographic* factors influencing hospital attendance rates when exposure to air pollution, where biological sex (female) and age (>65 years old) in all but one study (22); the latter demonstrated higher hospital attendance among those <60 years. In this study, the authors hypothesised that older adults may have paid more attention to preventing environmental hazards from PM pollution, such as staying at home during heavy pollution days (22). Older adults have a higher likelihood of being multi-morbid, with an international meta-analysis showing a marked increase in multimorbidity with increasing age (45). The present review highlighted how multi-morbid patients aged ≥70 years showing a 3.9% increase in cardiopulmonary hospital attendance, with short-term exposure to PM_2.5_, SO_2_, NO_2_, O_3_, or CO. (27) Furthermore, older adults aged +75 years had higher pneumonia admission prevalence with exposure to PM_10_ (26). A second large meta-analysis also found that females had a significantly higher prevalence of multimorbidity compared to males, particularly in the last two decades (46). This finding was largely identified in Europe, and South and North America (46). A Canadian study also found an increased prevalence of multimorbidity among elderly females of 75.9% in 2003 and 82.1% in 2016, compared to 72.5 and 75.4% in males, respectively (47). There are also physiological differences in inflammatory responses, lung size and blood-gas permeability amongst males and females (48). This increased prevalence of both multimorbidity and older female population, and potential physiological differences, could have contributed to the increased hospital attendance revealed in this review among this vulnerable group.

We found that *climatic* factors, such as temperature, can affect the levels of air pollutants, with high temperatures increasing the levels of PM or O_3_. This, in turn, can contribute to increased hospital attendance (49). In our review, 10 studies collected information about temperature and/or relative humidity (18–27), and adjustments made for these confounding variables (19–23, 25–27). However, only one study showed a strong correlation between PM_2.5_, PM_10_, SO_2_, CO and relative humidity (correlation coefficient > 0.80) (19). O_3_, average temperature and other pollutants showed a negative correlation (19). Another study in this review showed that all air pollutants (except O_3_) were strongly correlated with each other (coefficients distributed from 0.49 to 0.87) and moderately correlated with temperature and relative humidity (coefficients distributed from − 0.41 to 0.33) (22). In terms of the wider literature, Areal et al. (50) demonstrated an increased rate of respiratory hospital attendance and mortality with high temperature and exposure to PM_2.5,_ PM_10_, NO_2_ and O_3_. These findings highlight a potential gap in the literature in examining the associations between changes in temperature and relative humidity on hospital attendance among multimorbid patients. Furthermore, all included studies in this review were conducted in cool temperate climatic regions, Climate zones (51) and their findings cannot be extrapolated to countries in tropical or desert climatic zones (i.e., Africa and gulf area). This is a knowledge gap which would also need to be explored. The socio-economic level of a region could also impact the levels of air pollution; only one study provided information on the socio-economic level (i.e., 15% of its cohort were from deprived areas) in this review and this finding would also require further exploration (18).

To our knowledge, this was the first systematic review that looked at how different air pollutants were associated with hospital attendance in multimorbid patients. The use of multimorbidity groups in our review helped identify which long-term conditions can be affected by different air pollutions, and the rate of hospital attendance with exposure to these different air pollutants. However, some of the included studies in this review used fixed air quality monitoring tools, which might not have accurately recorded air pollution levels in certain areas. Additionally, some of the included studies did not adjust for the effect of confounders, such as temperature, age, occupation or social factors (i.e., smoking). It was inapplicable to conduct a meta-analysis for the included studies due to the heterogeneity of outcomes among the included studies (e.g., not all the studies included odd ratios to express hospital attendance). Additionally, the types of pollutants examined in the different studies were different, and the length of exposures were different.

## Conclusion

Short term (1–2 days) and long term (up to 30 years) exposure to air pollutants can increase the likelihood of hospital attendance and healthcare utilisation among multimorbid patients, especially older female adults. Many different factors can impact hospital attendance, described here as the “CADC” effect. These findings could inform governmental policies about finite hospital resources and promote person-centred clinical care. Future research is needed to explore this CADC effect particularly among underserved and deprived populations. Also, further research is needed in areas from different climatic zones to better understand the CADC effect.

## Data Availability

The original contributions presented in the study are included in the article/Supplementary material, further inquiries can be directed to the corresponding authors.
