# Supplementary material for: The impact of poor air quality on hospital attendance of multimorbid patients
Source: Front Med (Lausanne). 2026 Jan 7;12:1704117. doi: 10.3389/fmed.2025.1704117 (PMC12819827; doi:10.3389/fmed.2025.1704117)
Supplement: Supplementary file 1 [file Table_1.docx]

**Appendix 1- Search terms and strategy used in the systematic review for Poor Air Quality and Multimorbidity**

1. **Search Strategy and criteria used for Medline and Embase:**
   1. air pollution.mp. or exp Air Pollution/
   2. particulate matter.mp. or exp Particulate Matter/
   3. carbon monoxide.mp. or exp Carbon Monoxide/
   4. sulphur dioxide.mp. or Sulphur Dioxide/
   5. exp Air Pollutants/ or exp Sulfur Dioxide/ or sulphur dioxide.mp.
   6. nitrogen dioxide.mp. or exp Nitrogen Dioxide/
   7. ozone.mp. or exp Ozone/
   8. exp Air Pollutants/
   9. 1 or 2 or 3 or 5 or 6 or 7 or 8
   10. associated condition*.mp.
   11. associated diagnos*.mp.
   12. associated disease*.mp.
   13. associated health problem*.mp.
   14. associated illness*.mp.
   15. associated morbidity.mp.
   16. associated morbidities.mp.
   17. associated pathology.mp.
   18. associated pathologies.mp.
   19. coexisting condition*.mp.
   20. co-existing condition*.mp.
   21. co-existing disease*.mp.
   22. coexisting health problem*.mp.
   23. co-existing health problem*.mp.
   24. coexisting illness*.mp.
   25. co-existing illness*.mp.
   26. coexisting m bid*.mp.
   27. co-existing m bid*.mp.
   28. comorbid condition*.mp.
   29. co-morbid condition*.mp.
   30. comorbid disease*.mp.
   31. co-morbid disease*.mp.
   32. comorbid health problem*.mp.
   33. co-morbid health problem*.mp.
   34. comorbid illness*.mp.
   35. co-morbid illness*.mp.
   36. concurrent condition.mp.
   37. concurrent conditions.mp.
   38. concurrent disease*.mp.
   39. concurrent health problem*.mp.
   40. concurrent illness*.mp.
   41. concurrent m bid*.mp.
   42. cooccurring condition*.mp.
   43. co-occurring condition*.mp.
   44. co-occurring diagnos*.mp
   45. cooccurring disease*.mp.
   46. co-occurring disease*.mp
   47. cooccurring health problem*.mp.
   48. co-occurring health problem*.mp.
   49. cooccurring illness*.mp.
   50. co-occurring illness*.mp.
   51. co-occurring m bid*.mp.
   52. multiple condition.mp.
   53. multiple conditions.mp.
   54. multiple diagnosis.mp.
   55. multiple diagnoses.mp.
   56. multiple disease.mp.
   57. multiple diseases.mp.
   58. multiple health problem*.mp.
   59. multiple illness*.mp.
   60. multiple m bid*.mp.
   61. multiple pathology.mp.
   62. multiple pathologies.mp.
   63. Comorbidity.mp. or Comorbidity/
   64. comorbidities.mp.
   65. co-morbidity.mp.
   66. co-morbidities.mp.
   67. multidisease.mp.
   68. multi-disease.mp.
   69. multi-diseases.mp.
   70. multimorbidity.mp.
   71. multimorbidities.mp.
   72. multi-morbidity.mp.
   73. multi-morbidities.mp.
   74. multipathology.mp.
   75. multipathologies.mp.
   76. multi-pathology.mp.
   77. multi-pathologies.mp.
   78. pluripathology.mp.
   79. pluripathologies.mp.
   80. polypathology.mp.
   81. polypathologies.mp.
   82. poly-pathology.mp.
   83. poly-pathologies.mp.
   84. 10 or 11 or 12 or 13 or 14 or 15 or 16 or 17 or 18 or 19 or 20 or 21 or 22 or 23 or 24 or 25 or 26 or 27 or 28

or 29 or 30 or 31 or 32 or 33 or 34 or 35 or 36 or 37 or 38 or 39 or 40 or 41 or 42 or 43 or 44 or 45 or 46 or 47 or

48 or 49 or 50 or 51 or 52 or 53 or 54 or 55 or 56 or 57 or 58 or 59 or 60 or 61 or 62 or 63 or 64 or 65 or 66 or 67

or 68 or 69 or 70 or 71 or 72 or 73 or 74 or 75 or 76 or 77 or 78 or 79 or 80 or 81 or 82 or 83

- 1. hospital.mp. or Hospitals/
  2. inpatient.mp. or Inpatients/
  3. in-patient.mp.
  4. hospitalisation.mp.
  5. hospitalization.mp. or Hospitalization/
  6. hospitalised.mp.
  7. hospitalized.mp.
  8. 85 or 86 or 87 or 88 or 89 or 90 or 91
  9. 9 and 84 and 92

1. **Search Strategy and criteria used for Web of Science:**

“Air pollution” OR “Particulate matter” OR “PM10” OR “PM2.5” OR “ozone” OR “nitrogen dioxide” OR “sulphur dioxide” OR “carbon monoxide”

AND

“Hospital” OR “Hospitals” OR “Inpatient” OR “Inpatients” OR “In-patient” OR“Hospitalisation” OR “Hospitalization” OR “Hospitalised” OR “Hospitalized”

AND

“associated condition*” OR “associated diagnos*” OR “associated disease*” OR “associated health problem*” OR “associated illness*” OR “associated morbidity” OR “associated morbidities” OR “associated pathology” OR “associated pathologies” OR “coexisting condition*” OR “co-existing condition*” OR “co-existing disease*” OR “coexisting health problem*” OR “co-existing health problem*” OR “coexisting illness*” OR “co-existing illness*” OR “comorbid condition*” OR “co-morbid condition*” OR “comorbid disease*” OR “co-morbid disease*” OR “comorbid health problem*” OR “co-morbid health problem*” OR “comorbid illness*” OR “co-morbid illness*” OR “concurrent condition” OR “concurrent conditions” OR “concurrent disease*” OR “concurrent health problem*” OR “concurrent illness*” OR “concurrent m bid*” OR “cooccurring condition*” OR “co-occurring condition*” OR “co-occurring diagnos*” OR “cooccurring disease*” OR “co-occurring disease*” OR “cooccurring health problem*” OR “co-occurring health problem*” OR “cooccurring illness*” OR “co-occurring illness*” OR “co-occurring m bid*” OR “multiple condition” OR “multiple conditions” OR “multiple diagnosis” OR “multiple diagnoses” OR “multiple disease” OR “multiple diseases” OR “multiple health problem” OR “multiple illness*” OR “multiple m bid*” OR “multiple pathology” OR “multiple pathologies” OR “Comorbidity” OR “comorbidities” OR “co-morbidity” OR “co-morbidities” OR “multidisease” OR “multi-disease” OR “multi-diseases” OR “multimorbidity” OR “multimorbidities” OR “multi-morbidity” OR “multi-morbidities” OR “multipathology” OR “multipathologies” OR “multi-pathology” OR “multi-pathologies” OR “pluripathology” OR “pluripathologies” OR “polypathology” OR “polypathologies” OR “poly-pathology” OR “poly-pathologies”

1. **Search Strategy and criteria used for CINAHL:**

S1. “Air pollution” OR “Particulate matter” OR “PM10” OR “PM2.5” OR “ozone” OR “nitrogen dioxide” OR “sulphur dioxide” OR “carbon monoxide”

S2. “Hospital” OR “Hospitals” OR “Inpatient” OR “Inpatients” OR “In-patient” OR “Hospitalisation” OR “Hospitalization” OR “Hospitalised” OR “Hospitalized”

S3. “associated condition*” OR “associated diagnos*” OR “associated disease*” OR “associated health problem*” OR “associated illness*” OR “associated morbidity” OR “associated morbidities” OR “associated pathology” OR “associated pathologies” OR “coexisting condition*” OR “co-existing condition*” OR “co-existing disease*” OR “coexisting health problem*” OR “co-existing health problem*” OR “coexisting illness*” OR “co- existing illness*” OR “comorbid condition*” OR “co-morbid condition*” OR “comorbid disease*” OR “co-morbid disease*” OR “comorbid health problem*” OR “co-morbid health problem*” OR “comorbid illness*” OR “co- morbid illness*” OR “concurrent condition” OR “concurrent conditions” OR “concurrent disease*” OR “concurrent health problem*” OR “concurrent illness*” OR “concurrent m bid*” OR “cooccurring condition*” OR “co-occurring condition*” OR “co-occurring diagnos*” OR “cooccurring disease*” OR “co-occurring disease*” OR “cooccurring health problem*” OR “co-occurring health problem*” OR “cooccurring illness*” OR “co-occurring illness*” OR “co-occurring m bid*” OR “multiple condition” OR “multiple conditions” OR “multiple diagnosis” OR “multiple diagnoses” OR “multiple disease” OR “multiple diseases” OR “multiple health problem” OR “multiple illness*” OR “multiple m bid*” OR “multiple pathology” OR “multiple pathologies” OR “Comorbidity” OR “comorbidities” OR “co-morbidity” OR “co-morbidities” OR “multidisease” OR “multi-disease” OR “multi-diseases” OR “multimorbidity” OR “multimorbidities” OR “multi-morbidity” OR “multi-morbidities” OR “multipathology” OR “multipathologies” OR “multi-pathology” OR “multi-pathologies” OR “pluripathology” OR “pluripathologies” OR “polypathology” OR “polypathologies” OR “poly-pathology” OR “poly-pathologies” S4. S1 AND S2 AND S3

1. **Search Strategy and criteria used for Global Health:**

("Air pollution" OR "Particulate matter" OR "PM10" OR "PM2.5" OR "ozone" OR "nitrogen dioxide" OR "sulphur dioxide" OR "carbon monoxide") AND ("Hospital" OR "Hospitals" OR "Inpatient" OR "Inpatients" OR "In-patient" OR "Hospitalisation" OR "Hospitalization" OR "Hospitalised" OR "Hospitalized" ) AND ("associated condition*" OR "associated diagnos*" OR "associated disease*" OR "associated health problem*" OR "associated illness*" OR "associated morbidity" OR "associated morbidities" OR "associated pathology" OR "associated pathologies" OR "coexisting condition*" OR "co-existing condition*" OR "co-existing disease*" OR "coexisting health problem*" OR "co-existing health problem*" OR "coexisting illness*" OR "co-existing illness*" OR "comorbid condition*" OR "co-morbid condition*" OR "comorbid disease*" OR "co-morbid disease*" OR "comorbid health problem*" OR "co-morbid health problem*" OR "comorbid illness*" OR "co-morbid illness*" OR "concurrent condition" OR "concurrent conditions" OR "concurrent disease*" OR "concurrent health problem*" OR "concurrent illness*" OR "concurrent m bid*" OR "cooccurring condition*" OR "co-occurring condition*" OR "co-occurring diagnos*" OR "cooccurring disease*" OR "co-occurring disease*" OR "cooccurring health problem*" OR "co-occurring health problem*" OR "cooccurring illness*" OR "co-occurring illness*" OR "co- occurring m bid*" OR "multiple condition" OR "multiple conditions" OR "multiple diagnosis" OR "multiple diagnoses" OR "multiple disease" OR "multiple diseases" OR "multiple health problem" OR "multiple illness*" OR "multiple m bid*" OR "multiple pathology" OR "multiple pathologies" OR "Comorbidity" OR "comorbidities" OR "co-morbidity" OR "co-morbidities" OR "multidisease" OR "multi-disease" OR "multi-diseases" OR "multimorbidity" OR "multimorbidities" OR "multi-morbidity" OR "multi-morbidities" OR "multipathology" OR "multipathologies" OR "multi-pathology" OR "multi-pathologies" OR "pluripathology" OR "pluripathologies" OR "polypathology" OR "polypathologies" OR "poly-pathology" OR "poly-pathologies")

1. **Search Strategy and criteria used for Scopus:**

“Air pollution” OR “Particulate matter” OR “PM10” OR “PM2.5” OR “ozone” OR “nitrogen dioxide” OR “sulphur dioxide” OR “carbon monoxide”

AND

“Hospital” OR “Hospitals” OR “Inpatient” OR “Inpatients” OR “In-patient” OR “Hospitalisation” OR “Hospitalization” OR “Hospitalised” OR “Hospitalized”

AND

“Comorbidity” OR “comorbidities” OR “co-morbidity” OR “co-morbidities” OR “multidisease” OR “multi-disease” OR “multi-diseases” OR “multimorbidity” OR “multimorbidities” OR “multi-morbidity” OR “multi-morbidities” OR “multipathology” OR “multipathologies” OR “multi-pathology” OR “multi-pathologies” OR “pluripathology” OR “pluripathologies” OR “polypathology” OR “polypathologies” OR “poly-pathology” OR “poly-pathologies”
