# Supplementary material for: The impact of poor air quality on hospital attendance of multimorbid patients
Source: Front Med (Lausanne). 2026 Jan 7;12:1704117. doi: 10.3389/fmed.2025.1704117 (PMC12819827; doi:10.3389/fmed.2025.1704117)
Supplement: Supplementary file 4 [file Table_4.docx]

Newcastle Ottawa Scale for risk of bias assessment.

| **Study** | **Selection of cohorts** | | | | | **Comparability of cohorts** | | **Outcome** | | | | **Total Score** | | **Quality** | |
| --- | --- | --- | --- | --- | --- | --- | --- | --- | --- | --- | --- | --- | --- | --- | --- |
|  | Representativeness of the exposed cohort | Selection of the non exposed cohort | Ascertainment of exposure | Demonstration that outcome of interest was not present at | Comparability of cohorts on the basis of the design or analysis | | Assessment of outcome | | Was follow up long enough for outcomes to occur | Adequacy of follow up of cohorts |  | |  | |  |
| Chen et al. (17) | 1 | 1 | 1 | 1 | 2 | | 1 | | 1 | 1 | 9 | | Good | |  |
| Li et al. (18) | 1 | 1 | 1 | 1 | 1 | | 1 | | 1 | 1 | 8 | | Good | |  |
| Lin et al (19) | 1 | 1 | 1 | 1 | 1 | | 1 | | 1 | 1 | 8 | | Good | |  |
| Liu et al. (20) | 1 | 1 | 1 | 0 | 2 | | 1 | | 0 | 1 | 7 | | Fair | |  |
| Liu et al. (21) | 1 | 1 | 1 | 1 | 1 | | 1 | | 1 | 1 | 8 | | Good | |  |
| Chen et al.(22) | 1 | 1 | 1 | 1 | 2 | | 1 | | 1 | 1 | 9 | | Good | |  |
| Seposos et al. (23) | 1 | 1 | 1 | 0 | 2 | | 1 | | 1 | 1 | 8 | | Good | |  |
| Liu et al. (24) | 1 | 1 | 1 | 1 | 2 | | 1 | | 1 | 1 | 9 | | Good | |  |
| Wang et al. (25) | 1 | 1 | 1 | 1 | 2 | | 1 | | 1 | 1 | 9 | | Good | |  |
| Lee et al. (26) | 1 | 1 | 1 | 1 | 1 | | 1 | | 1 | 1 | 8 | | Good | |  |
| Cheng et al. (27) | 1 | 1 | 1 | 1 | 1 | | 1 | | 1 | 1 | 8 | | Good | |  |
| Zhang et al.(28) | 1 | 1 | 1 | 1 | 2 | | 1 | | 1 | 1 | 9 | | Good | |  |
| Alvaro-Meca et al. (29) | 1 | 1 | 1 | 1 | 2 | | 1 | | 1 | 1 | 9 | | Good | |  |
| Carlsen et al (30) | 1 | 1 | 1 | 1 | 2 | | 1 | | 1 | 1 | 9 | | Good | |  |
| Andersen et al | 1 | 1 | 1 | 1 | 2 | | 1 | | 1 | 1 | 9 | | Good | |  |
| Cheng et al. (32) | 1 | 1 | 1 | 1 | 2 | | 1 | | 1 | 1 | 9 | | Good | |  |
| Peel et al. (33) | 1 | 1 | 1 | 1 | 2 | | 1 | | 1 | 1 | 9 | | Good | |  |
| D'Ippoliti et al. (34) | 1 | 1 | 1 | 1 | 2 | | 1 | | 1 | 1 | 9 | | Good | |  |
| Zanobetti et al. (35) | 1 | 1 | 1 | 1 | 1 | | 1 | | 1 | 1 | 8 | | Good | |  |

Thresholds for converting the Newcastle-Ottawa scales to AHRQ standards (good, fair, and poor):

**Good quality:** 3 or 4 stars in selection domain AND 1 or 2 stars in comparability domain AND 2 or 3 stars in outcome/exposure domain

**Fair quality:** 2 stars in selection domain AND 1 or 2 stars in comparability domain AND 2 or 3 stars in outcome/exposure domain

**Poor quality:** 0 or 1 star in selection domain OR 0 stars in comparability domain OR 0 or 1 stars in outcome/exposure domain
